# Langerhans Cells—Programmed by the Epidermis

**DOI:** 10.3389/fimmu.2017.01676

**Published:** 2017-11-29

**Authors:** Kalum Clayton, Andres F. Vallejo, James Davies, Sofia Sirvent, Marta E. Polak

**Affiliations:** ^1^Systems Immmunology Group, Clinical and Experimental Sciences, Sir Henry Wellcome Laboratories, Faculty of Medicine, University of Southampton, Southampton, United Kingdom

**Keywords:** Langerhans cells, transcription factors, gene regulatory networks, epidermis, immune regulation, dendritic cells, macrophages, cross-presentation

## Abstract

Langerhans cells (LCs) reside in the epidermis as a dense network of immune system sentinels. These cells determine the appropriate adaptive immune response (inflammation or tolerance) by interpreting the microenvironmental context in which they encounter foreign substances. In a normal physiological, “non-dangerous” situation, LCs coordinate a continuous state of immune tolerance, preventing unnecessary and harmful immune activation. Conversely, when they sense a danger signal, for example during infection or when the physical integrity of skin has been compromised as a result of a trauma, they instruct T lymphocytes of the adaptive immune system to mount efficient effector responses. Recent advances investigating the molecular mechanisms underpinning the cross talk between LCs and the epidermal microenvironment reveal its importance for programming LC biology. This review summarizes the novel findings describing LC origin and function through the analysis of the transcriptomic programs and gene regulatory networks (GRNs). Review and meta-analysis of publicly available datasets clearly delineates LCs as distinct from both conventional dendritic cells (DCs) and macrophages, suggesting a primary role for the epidermal microenvironment in programming LC biology. This concept is further supported by the analysis of the effect of epidermal pro-inflammatory signals, regulating key GRNs in human and murine LCs. Applying whole transcriptome analyses and *in silico* analysis has advanced our understanding of how LCs receive, integrate, and process signals from the steady-state and diseased epidermis. Interestingly, in homeostasis and under immunological stress, the molecular network in LCs remains relatively stable, reflecting a key evolutionary need related to tissue localization. Importantly, to fulfill their key role in orchestrating antiviral adaptive immune responses, LC share specific transcriptomic modules with other DC types able to cross-present antigens to cytotoxic CD8^+^ T cells, pointing to a possible evolutionary convergence mechanism. With the development of more advanced technologies allowing delineation of the molecular networks at the level of chromatin organization, histone modifications, protein translation, and phosphorylation, future “omics” investigations will bring in-depth understanding of the complex molecular mechanisms underpinning human LC biology.

## Introduction

**Figure 1:**
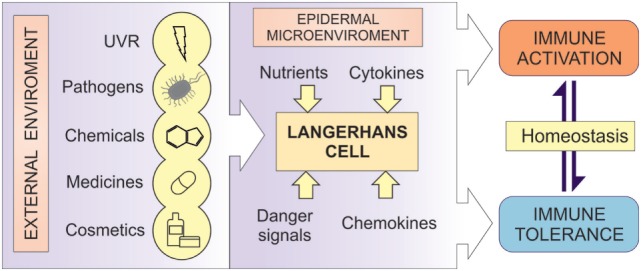
LCs as the regulators of immune responses. A diagrammatic representation of the central role of LCs in human epidermis. LCs act as cellular transducers, transmitting signals encountered at the epidermal surface, including ultraviolet radiation (UVR), chemicals, cosmetics, pathogens, and medicines, as well as signals from the microenvironmental compartment in control of immune homeostasis. In disease state, LC function can be modified by the aberrant signaling from both the environment and the microenvironment, resulting in altered immune regulation in inflammatory disorders.

One of the most critical functions of the skin required by its role as the interface with the external environment, is to defend against microbial attack. This antimicrobial defensive function is achieved through the double mechanisms of both the innate and the adaptive immune responses (1). One of the key cellular components with functional roles in both innate and adaptive arms of the immune response are Langerhans cells (LCs) (2). LCs are members of the dendritic cell (DC)/macrophage family, and they reside in the epidermis, forming a dense network with which potential invaders must interact. LCs are uniquely specialized at “sensing” the environment, extending dendritic processes through intercellular tight junctions to sample the outermost layers of the skin (stratum corneum) (3). They interpret the microenvironmental context in which they encounter foreign proteins and, hence, determine the appropriate quality of the immune response. Under quiescent (non-dangerous) conditions, LCs selectively promote expansion and activation of skin-resident regulatory T cells (Tregs) (4, 5). However, when LC are perturbed by “sensing” danger in the form of microbial components, together with the epidermal keratinocytes, they participate in rapid innate antimicrobial responses but critically, they also initiate the power and specificity of the T cell components of the adaptive response (4, 6, 7) (Figure 1). LC function can be profoundly modified by cytokine signals from structural cells of the epidermis, such as keratinocytes, resulting in alteration of the type of adaptive immune responses induced. In particular, tumor necrosis factor-α (TNF-α), which plays an important role in the initiation and persistence of inflammation in a variety of skin disorders, can potently stimulate LCs, inducing their activation (8–10), *in situ* motility and pathogen sensing (11), and antigen presentation (12, 6). Cytokines released by keratinocytes in atopic dermatitis, e.g., thymic stromal lymphopoietin (TSLP), alter LC’s ability to induce adaptive immune responses (13, 14), while “homeostatic” cytokines, such as TGF-β, inhibit LC maturation *in situ* and are critical for LC retention in the epidermis (15, 16).

**Figure 2:**
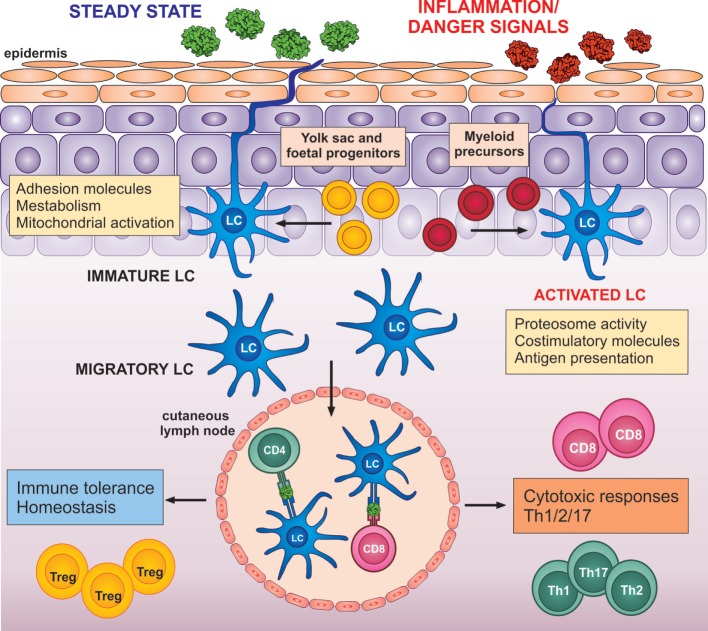
LC transition: from a steady state to potent adaptive immune activators. LCs are seeded in the epidermis from yolk sac and fetal liver progenitors (yellow). Only in the case of severe local inflammation, LCs could be repopulated by local blood progenitors (red). LCs in the steady state in the epidermis express high levels of adhesion molecules, such as E-cadherin and proteins, involved in metabolism and mitochondrial activation. Upon migration from the epidermis, LCs increase expression of co-stimulatory molecules, proteosome activity, and antigen presenting molecules. Upon detection of danger signals, LC activation is enhanced and altered by signals from the inflammed epidermis. Both, steady-state or activated LCs can migrate to drained lymph nodes, where they instruct the adaptive immune system toward immune tolerance (left) or immune activation (right).

In contrast to many DC subtypes derived directly from a myeloid progenitor, signaling from the epidermis uniquely shapes both the function and the development of LCs from the earliest stages of ontogeny. The heterogeneous human LC progenitors appear at about 7 weeks of gestational age and establish the skin LC pool (17). Subsequently, under the steady state, scattered proliferative precursors self-renew *in situ* at a very low rate, without any influx of circulating precursors (18–21). Only in rare instances, when a severe local inflammation induces LC depletion, inflamed-state LCs would reconstitute the LC compartment, either in transient or stable manner (22) (Figure 2).

The critical importance of the LC’s ability to discriminate between signals that indicate danger and those which are non-threatening is reflected by the immunological tolerance that prevents unwanted immune-mediated reactions to routinely encountered environmental substances. Among the best recognized examples are the non-reactivity (tolerance) to nickel, encountered daily through contact with metal objects, or as a result of interactions with symbiotic microorganisms inhabiting the skin (4, 5), and mediated by tolerogenic T cells (23, 24). However, when danger signals are provided through, for example, tissue damage from ear-piercing (nickel) or from microbial components signaling through toll-like receptors, the immune system generates active effector T cells required for protective immunity (4, 23). Similarly, exposure to ultraviolet radiation alters the epidermal microenvironment so that danger signals are ineffective and chemical contact sensitizers such as dinitrochlorobenzene (DNCB) induce immunological tolerance (25, 26). Furthermore, keratinocytes, the structural cells of the epidermis, provide signals at the time of sensitization, which modify the nature of the induced adaptive immune responses, and the polarization of the antigen-specific T lymphocytes, e.g., inducting long-lasting DNCB-specific Th2 responses in atopic individuals (27).

This ability of LCs to shape the outcome of the local and systemic immune responses can be harnessed, for example, in LC-mediated immunotherapeutic interventions. While sub-cutaneous allergen-specific immunotherapy (without adjuvant immunostimulation) induces antigen-specific tolerance (28, 29), transcutaneous vaccination with adjuvant immunostimulation (danger), requires only one-fifth of the dose of antigen to induce systemic protection levels comparable with classical intramuscular administration (30, 31).

Despite the paramount importance for human body homeostasis and the generation of appropriate immune responses, our understanding of this decision-making process is lacking. Limited availability of human LCs and the technical constrains of experimental models are major limiting factors. LCs are relatively infrequent in skin, isolation of LCs is laborious and requires sufficiently large amounts of skin tissue to obtain adequate cell numbers for functional analysis. As a result, many models of human LCs have been used, such as monocyte-derived DCs, but possibly due to the limitations of the models employed, controversies exist about how well they reflect LC function. Furthermore, it is not always possible to extrapolate data from murine epidermis into understanding of human LCs biology, as conflicting experimental results indicate they may play different roles in regulation of human and murine cutaneous immune responses (7, 6, 32–36).

The advent of high throughput (such as microarrays) and next-generation (such as RNA-sequencing) omics technologies offers unprecedented opportunity to investigate LCs in detail at the whole transcriptome level. These approaches provide insights into the molecular mechanisms underpinning LC biological function and identify molecular switches controlling the transcriptomics networks orchestrating it. This review aims to reflect the novel insights, which the power of the “omics” technologies has provided on the important questions regarding human LC origin, classification, and function.

## Langerhans’ Cells: DCs or Macrophages?—View from the Transcriptome

Tissue-resident antigen-presenting cells can be classified into two types: DCs and macrophages. While macrophages are the core phagocytes and activators of the innate immune system, DCs represent a small population of hematopoietic antigen-presenting cells that have the unique ability to prime naïve T lymphocytes. DCs share some properties with tissue macrophages. These include their localization in most tissues, sensing environmental “insults” and injuries through their ability to sample extracellular antigens and contributing to the induction of tissue immune responses (37). Following migration to the lymph nodes, DCs are able to prime adaptive immune responses inducing either activation or tolerance. DCs express lymphocyte co-stimulatory molecules and secrete cytokines; the array of these signals determines the subsequent outcome of adaptive immunity. DCs can be subdivided into two major types: myeloid and plasmacytoid DCs (pDCs). These two DC types are characterized by divergent antigen processing abilities and responses to immune stimuli, along with engaging different effector lymphocytes. Classical DCs (cDCs) form the predominant myeloid DC subset and can be further subcategorized as non-lymphoid or lymphoid tissue-resident cDCs. While pDCs can sense both bacterial and viral pathogens, they are thought to specialize in initiating antiviral cytotoxic T cell immunity and are uniquely able to produce large amounts of the antiviral cytokine interferon-α (38).

**Figure 3:**
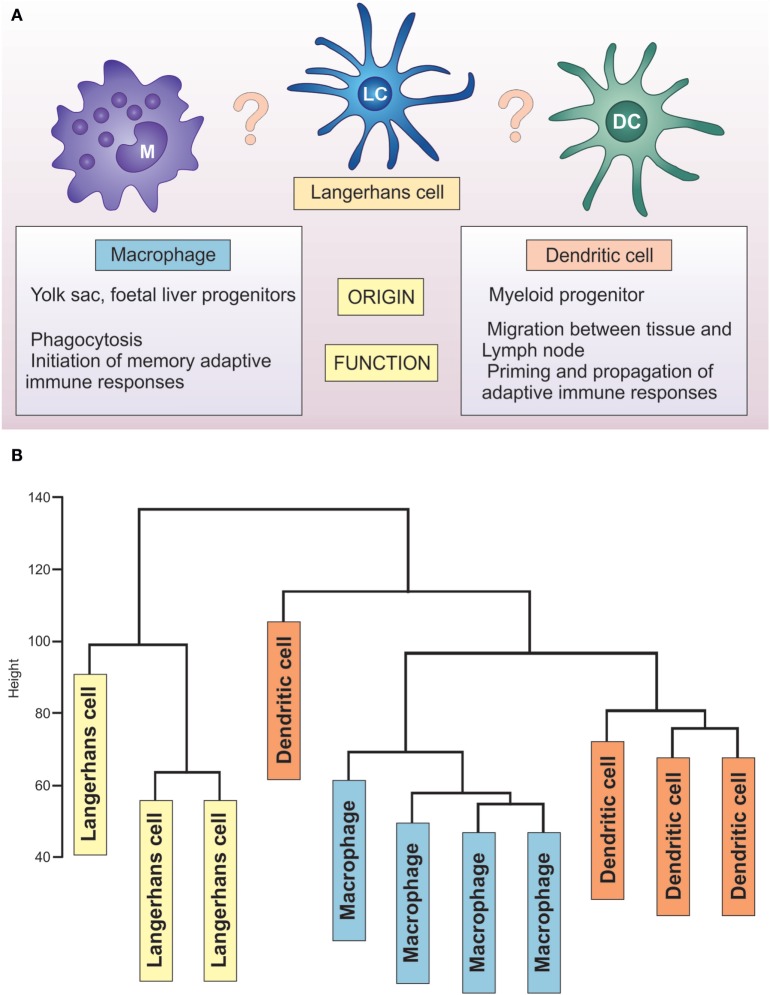
Unique transcriptome of LCs distinguishes them from DCs and macrophages. **(A)** LCs share characteristics with macrophages (yolk sac origin, tissue residence) and DCs (priming T lymphocytes, migration to the lymph nodes). **(B)** Hierarchical clustering analysis of GSE60317 dataset. Samples include LCs (yellow box), CD14^+^ dermal DCs (orange box), and cutaneous macrophages (blue box) total RNA expression data. Data obtained from GEO (Illumina Human WG-6 v3.0) were log transformed and normalized before using all probes for hierarchical clustering analysis (hclust package, R: Euclidean distance and complete clustering). The cluster analysis revealed LCs to be transcriptionally distinct from both dermal CD14^+^ dendritic cells and macrophages.

Placing LCs within this spectrum is not trivial, as recently reviewed by Doebel and colleagues (39) (Figure 3). Identified in 1868 by a medical student, Paul Langerhans, they were first regarded as being related to nerve cells because of their “dendritic” morphology. It was not until the 1970s, when Silberberg et al. showed they formed close contacts with lymphocytes, that an immune function was considered (40). For some time, they were considered as prototypic DC, key to initiation of CHS. With the recent understanding of their ontogeny, self-renewal abilities, and the life-long localization in the epidermis, they have been considered to be a specialized subset of tissue-resident macrophages (39). In contrast, a significant body of work on LC functional properties documents that, similar to DCs, they migrate to lymph nodes and present antigen to antigen-specific T cells (41–43) (Figure 3).

Analysis of transcriptome-wide gene expression in LCs brings new insights to this long-standing controversy. An extensive, direct comparison of three key antigen-presenting cell populations (LCs, DCs and macrophages) across 87 samples from the human vagina, skin, and blood, performed by Duluc and colleagues, characterized the differences between the subsets at the level of whole transcriptome gene expression (44). The study highlighted the critical role of the tissue environment in shaping the LC transcriptome (and thus programming the cell function). The main separation of the cell transcriptomes in the skin resulted from their localization in the epidermal and dermal compartments, in contrast to the vaginal mucosa, where transcriptionally, LCs and CD14^−^ DCs were very similar. Principal component analysis has clearly outlined that human vaginal LC cluster away from macrophages, together with CD14^−^ DC, faithfully to the tissue, not to the cell subset. Epidermal LCs displayed a transcriptomic signature encoding pathways involved in immune regulation, while dermal CD14^−^ and CD14^+^ DCs displayed an innate immunity and pro-inflammatory profile akin to that of vaginal CD14^+^ APCs. The paramount importance of tissue microenvironment in shaping cell immune programming is corroborated by the studies of tissue-resident macrophages and DCs (45–47). Elegant studies in a murine system demonstrated that after complete replacement of the embryo-derived tissue macrophage compartment with adult blood-derived progenitors, the transplant-derived macrophages showed a phenotype more similar to their embryonic tissue-residing counterparts than to transplanted macrophages in other tissues (45). In humans, the effect of tissue microenvironment seems to be particularly important in the body surfaces in contact with the environment—such as skin and lung. Thus, DC subpopulations from those sites cluster in accordance with the tissue or origin, in contrast with subsets of DCs derived from lymphohematopietic system, defined by ontogeny (47).

Unfortunately, the dataset of Duluc et al. did not contain human skin macrophages, making the direct classification of human skin APC impossible. Some insights can be drawn from re-analysis of a publicly available data set deposited by Haniffa et al. (GEO 60317), containing human skin migratory APCs: CD14^+^ dermal DCs, LCs, and dermal macrophages. Hierarchical clustering of these three human skin APC subsets reveals a distinct LC cluster separated from both CD14^+^ dermal DCs and macrophages (Figure 3), confirming the uniqueness of epidermal LCs. This separation is preserved when put in comparison with monocytes, macrophages, and different populations of skin and blood DCs in a manner that is conserved across species (48). Indeed, the analyses performed by the Immunological Genome Consortium document that murine non-lymphoid LC clustered separately from eight other distinct tissue-resident DC populations, expressing only 50% of core cDC transcripts (49). Further evidence for the uniqueness of LC comes from two meta-analyses of publicly available datasets, which demonstrated LCs clustering away from the majority of other DC types, including monocytes and monocyte-derived cells (48, 50).

To fulfill their immunoregulatory role, LC leave the epidermis and migrate to the regional lymph node. An Immunological Genome Consortium study (49), characterized 26 distinct murine DC populations isolated from primary lymphoid tissues, secondary lymphoid tissues, and non-lymphoid tissues. Their analysis showed that the proximity of murine LC to the DC family changes significantly after they migrate into the lymphoid tissue. Interestingly, once LCs leave the epidermis, they upregulate Flt3 and they become significantly more similar to cDC, in particular to CD103^+^ migratory DCs (49), clustering mid-way between cDCs and macrophages. This specific transcriptional program was observed during the steady-state migration to the draining lymph nodes.

**Figure 4:**
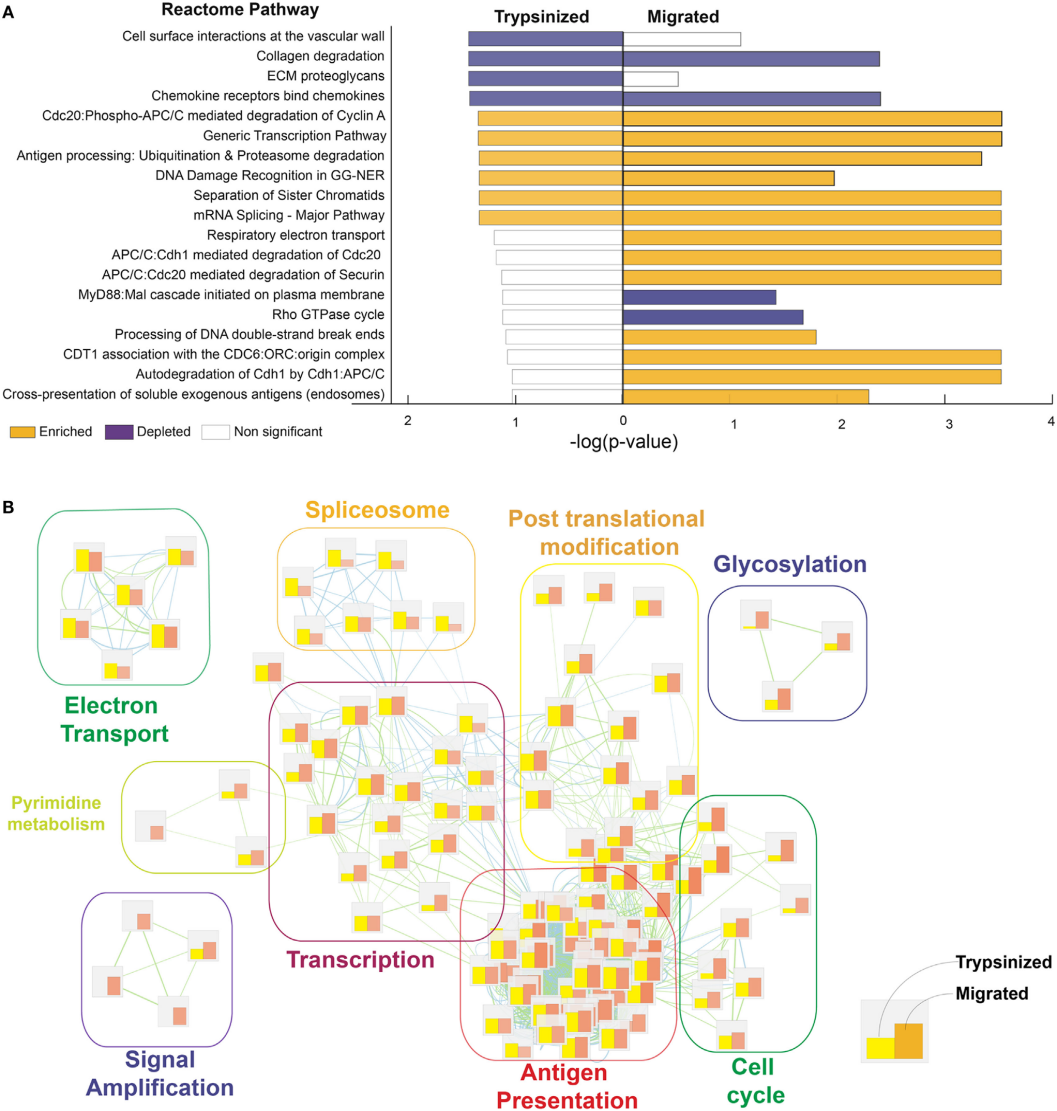
LC migration out of the epidermis enhances their ability to process and cross-present antigens. Processes regulated in trypsinazed “steady-state” versus migrated “activated” LCs. Corresponding dermal DCs were used as a baseline for each population, to enable cross-platform comparison. While both migratory and steady-state LCs presented significant enrichment in antigen presentation, DNA replication, and gene transcription in comparison to their corresponding dermal DCs, the differences are more pronounced for migratory (activated) LCs. **(A)** Gene ontology analysis was done using Genetrail2 using skin LCs and dermal DCs data sets downloaded from GEO (GSE49475, GSE23618). Processes enriched in of LCs versus dermal DCs were compared between trypsinazed and migrated cells using Reactome knowledge database. Enriched pathways (yellow bars), and depleted pathways (purple bars) shown, bar length denotes enrichment −log (*p*-value). **(B)** Enrichment Map representation of the GSEA results obtained for steady-state and activated LCs in comparison to dermal DC. Bars represents the normalized enrichment score for each process, yellow: trypsinised, orange: migratory LC. Over represented terms were obtained using world cloud plugin in Cystoscape.

Meta-analysis of human cutaneous LC, isolated by allowing them to migrate out of excised skin (migratory cells) (12) and cells extracted rapidly by trypsinisation (51) (previously published datasets derived from Gene Expression Omnibus, GSE49475, GSE23618), further supports the idea that the event of breaking from the epidermal microenvironment is critical for the transcriptional programming of LC function. Our comparisons indicate that human migratory LC acquires high T cell stimulatory abilities while retaining the main pattern of gene expression in a steady state (Figure 4). In particular, migratory LC gene expression is marked by reduced but not absent cell adhesion molecule expression, with increased cell metabolism, protein catabolism, and cytoskeletal rearrangement processes. Importantly, migration increases expression of genes involved in immune proteasome functions and increases expression of co-stimulatory molecules (40). In contrast, steady-state LCs were characterized by increased mitochondrial activation, a potential advantage for cells residing in a low nutrient, low oxygen, minimally vascularized tissue such as the epidermal compartment [(12), Figure 4]. Furthermore, antigen uptake by steady-state LCs can play a critical role in preventing viral infections, for example, the expression of the C-type lectin receptor, Langerin, facilitates the capture of HIV-1 to prevent infection by subsequent sequestration within LC Birbeck granules (52, 53).

The existing evidence indicates that, based on their transcriptome, LCs are distinct from both DCs and macrophages. This supports the concept that the epidermal microenvironment acting on LC from the stage of an early progenitor cell plays a critical role in shaping LC biology and tailors it uniquely for the requirements of the tissue they populate. Breaking free from the epidermal microenvironment is a transformative event in LC immunological activation, leading to the reorganization of the transcriptomic networks and initiation of antigen processing and presenting machinery.

## Human LC Preference for Precise T Cell Activation Revealed by Transcriptome Analyses

We and others have previously demonstrated that activated LCs turn on a very characteristic transcriptional program, producing very few typical inflammatory mediators, including low levels of IL-1β and IL-12p70 in comparison to their dermal counterparts (6, 36, 54, 55). In contrast, the ability of LCs to efficiently process and present antigens to CD4 and CD8 T cells underpins their potency in induction of Th2, Th17, regulatory, and humoral immune responses, and their critical role in initiation and maintenance of CD8 T cell immunity (4–7, 12, 36, 56–60).

The migration of LCs to the inner paracortex of the draining lymph node gives a clue to their effector function. Work by Klechevsky et al. (7) shows that LCs preferentially select and expand antigen-specific cytotoxic T cells. LCs have been shown to be better than dermal DCs at cross-presentation of viral antigens to IFN-gamma secreting CD8 T cells (12, 6, 36, 57). These data suggest that LCs are the primary professional APC and activators of cellular cytotoxic immunity in the skin, particularly for antiviral responses. Infections by human papilloma virus, herpes simplex virus, and HIV, which require potent cellular cytotoxic responses for effective immunity, all involve early infection of LCs by the virus (61–64). Recent reports that suggest antigen exchange interactions between LCs and dermal DC subsets do not diminish the crucial antiviral role of LCs, demonstrated in murine and human systems. For example, in HSV infection, which principally targets keratinocytes resulting in cell apoptosis (55), LC uptake and processing of HSV antigens from apoptotic KCs appears to be crucial for initiation of anti-HSV immune responses (62, 65).

The high functional capacity of LCs for MHC class I presentation and their potent ability to drive CD8 T cell responses is reflected in their transcriptional profile (12, 48, 66). Our work on the distinct transcriptomes of LCs and dermal CD11c^+^ DCs shows that the phenotypic and functional differences between them is both marked and driven at the transcriptional level. Gene ontology analysis demonstrates that migratory LCs are geared toward processes involved with or linked to antigen cross-presentation, such as endocytosis and intracellular transport, proteolysis, and mitochondrial and metabolic activity. The dichotomy of anatomical location of LCs is indeed matched by their function, which itself is driven by intrinsically distinct molecular signatures of their respective transcriptomes (12, 13). In contrast, LC share the specific transcriptional modules encoding proteins involved in antigen processing and cross-presentation with other DC types able to cross-present, including the mouse XCR1^+^ CD8a^+^ CD103^+^ DCs and human dermal CD141 DCs (48, 66, 67).

The distinctiveness of molecular networks in skin LC, and their preference for precise activation of antigen-specific adaptive immune responses over inflammation, strongly supports the concept that their biology is adapted to the specific requirements of the local tissue microenvironment (13, 44, 68, 69). LC transcriptomic programs can be explained as a direct result of differentiation from LC precursors being directed by interactions between LCs, structural cells of the epidermis, and the symbiotic microbiota during tissue-resident differentiation from LC precursors (22, 70) (Figure 2). Preventing over-activation and adverse inflammatory responses in the epidermis is critical. Unrestrained inflammation can potentially disrupt the skin barrier to allow entry of infectious agents into the body. The ability to maintain tissue homeostasis without causing adverse inflammatory responses by limiting presentation of bacterial antigens and inducing Tregs during steady-state conditions, therefore, seems to be one of the key functions of epidermis-resident steady-state LCs (4, 5, 71).

## Immune Activation Transcriptomic Programs in Human LCs are Orchestrated by Interferon Regulatory Factor (IRF) Gene Regulatory Network (GRN)

**Figure 5:**
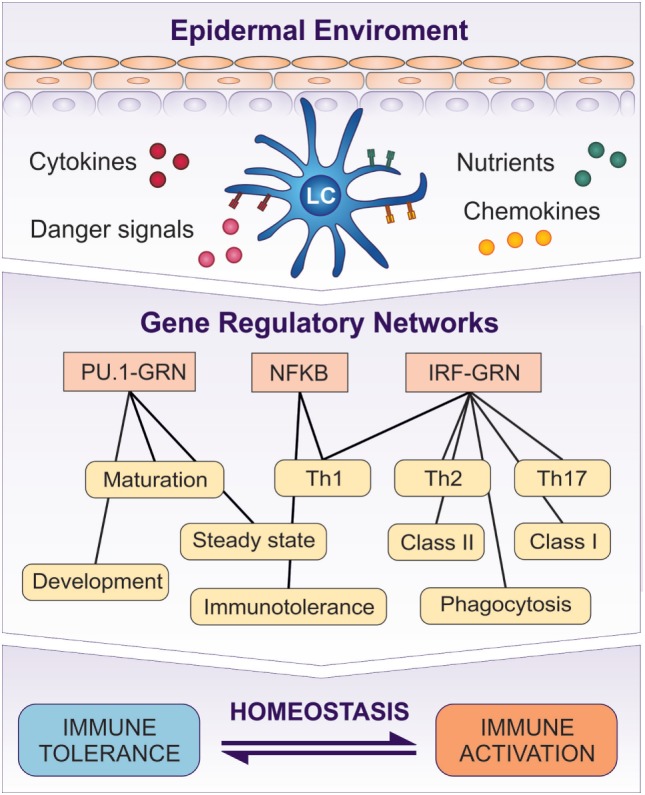
Transcriptomic programs initiated in LCs by the signaling from the epidermis are controlled by GRNs. PU.1-GRN controls LC development and steady state, while Interferon Regulatory Factor (IRF)–GRN regulates LC activation and antigen presentation in MHC class I and II. NF-κB system is involved in both induction of tolerance and activation. A switch between PU.1 controlled GRN and IRF–NF-κB–GRN regulates LC function, and determines whether it induces tolerance or immune activation.

Signals from the external environment and the epidermal microenvironment, such as cytokines, chemokines, pathogens, chemicals, and UV radiation, can potentially have profound effects in regulating LC immune programming (Figure 2). From the earliest stages of development, the ability to monitor and “interpret” correctly the received signals from the ever-changing epidermal microenvironment is a critical functional role of LCs. It is most unlikely that their functional responsiveness will be dependent on adaptations within a single protein or pathway but is much more likely to be controlled *via* a coordinated network of biochemical reactions mediated by any number of molecular species, underpinned by coordinated expression of RNA transcripts (Figure 5).

To describe and investigate the complexity of regulation of transcriptomic programs, the concept of “GRN” was introduced (72, 73). Within a GRN, coordinated expression of a required but diverse set of target genes (i.e., “transcriptomic programs”) is controlled by key transcription factors. These work in synergistic or antagonistic interactions with other transcription factors, adaptor molecules, and kinases, generating a plausible conceptual framework for this decision-making process (72, 74, 75).

Studies of the candidate transcription factors regulating human LC development and function clearly identify members of two interacting families: IRF (13, 42, 76, 77) and the NF-κB system (78–81). Our analysis and *in silico* modeling of the time course of changes in transcriptional networks in human LCs exposed to signaling from two epidermal cytokines, TNF-α and TSLP, confirmed that a set of transcription factors from the IRF family act as a key GRN operating in human LCs. This led to assembling a model of the IRF gene regulatory network (IRF–GRN) comprising *IRFs*, transcription partners, DNA sequences, and transcribed genes under the control of IRFs (13). Network simulation with the Stochastic Petri Net algorithm predicted the existence of two distinct transcriptional programs controlled by the IRF–GRN and regulating the ability of LCs to present antigens. Program “A” included genes preferentially induced by TNF-α after binding of transcription factors to Interferon-Stimulated Response Element. Program “B” comprised genes similarly regulated by TNF-α and TSLP, induced after transcription factor binding to ETS-IRF composite element. Thus, the epidermal derived cytokines TNF-α and TSLP altered the expression of genes associated with LC activation (CD40), antigen uptake and processing (CAV1, PSME1, PSME2, PSMB10), and antigen presentation (HLA-A, -B, -C, CIITA, HLA-DR), enhancing LC ability to activate antigen-specific adaptive immune responses and to cross-present antigens to CD8 T cells. This model strongly supports the importance of antigen processing and presentation for LC function and provides a molecular explanation for regulation of LC immune programming by signals from the epidermis (13). The differences in the cytokine milieu produced by healthy and atopic keratinocytes, mimicked by TNF-α and TSLP signaling, impact LC ability to process, present and cross-present antigens. In disease, such as atopic dermatitis, skin immunity against viral infection may be diminished and potentially contribute to the recurrent viral infections in eczema herpeticum.

As demonstrated recently, IRF-controlled GRNs coordinate transcriptional programs in a number of DC subsets in murine and human blood and spleen (72, 74, 75). DC development is critically regulated by IRF-4 and IRF8 molecules, which also manage proper co-ordination of immune responses to infectious pathogens (74, 82, 83), while IRF-3 and IRF7 have been implicated in induction of inflammatory responses and DC maturation (84, 85). While this makes the IRF–GRN an important element of LC/DC programming, a number of studies identify further candidate regulators, possibly acting in concert—or inhibiting the IRF–GRN in LCs.

## NF-κB System

NF-κB is classically regarded as central to the activation of immune responses. However, in LCs, signaling *via* the TNF superfamily member receptor activator of NF-κB (RANK) and its ligand, RANKL, mediates active immunotolerance. Epidermal expression of RANKL has a critical role regulating LC survival and suggests the maintenance of epidermal LC homeostasis is, in part, maintained by signals from local KCs (86). In healthy adult human epidermis, flow cytometry reveals that ~95% of LCs express RANK (86), while keratinocytes express low levels of RANKL. LC gradually acquire RANK during gestation, reaching levels comparable with adult skin in the third trimester of pregnancy—a phenomenon previously observed for other markers on LCs in prenatal human skin (87).

A similar signaling cascade seems to be activated in the immunosuppressive context that results from ultraviolet irradiation. Keratinocytes upregulate RANKL and trigger RANK expressing LCs (88) preferentially to expand the subset of CD4^+^ CD25^+^ Treg suppressive for local and systemic immune reactions (87). Furthermore, LCs stimulated with sRANKL have been shown to augment the expression of C–C motif chemokine ligand 17 (CCL17), and induce Foxp3^+^ Tregs (89).

In contrast, KC-RANKL: LC RANK signaling is simultaneously involved in promoting the ability of LC to activate effective adaptive immune responses in a variety of immunostimulatory situations, e.g., mediating IgE antibody signaling in donors expressing high FceRI levels on epidermal LC (79). Similarly, in mice, substance P activates LCs through NK1 receptor, causing translocation of NF-κB into the nuclei of cells homing to skin-draining lymph nodes. RANKL:RANK engagement prevents LC apoptosis, and enhances LC migration to regional lymph nodes. This is associated with an improved induction of MHC class I-restricted HSV-1-specific antiviral immunity, dependent on TLR3 signaling (90). We and others have shown that LCs are equipped with a range of surface receptors, including pattern recognition receptors, pathogen uptake-receptors (Langerin and DEC205) as well as intracellular sensors of microenvironmental changes, such as caveolin (CAV1) and endosulfine alpha (12, 60, 91–95). Activation of NF-κB seems to be important for many of these, e.g., ligation of TLRs with microbial PAMPs induces NF-κB-mediated upregulation of CCR7, CD86, CD83, TNF-α, and IL-6, and activation and proliferation of CD4 T cells (96).

The immunoactivatory NF-κB signaling can be intimately linked with the IRF network, as shown by Cheng et al. (97). Indeed, Wang and colleagues demonstrated that in human LCs, TLR 2 and 4 signal through NF-κB/p65 and IRF-3 (78). Similarly, such interactions between IRF and NF-κB signaling networks in coordinating the expression of various gene programs balancing the tolerogenic versus immunogenic function has been recently observed in cDCs (84, 98). Here, the postulated switch between tolerance activation lies within NF-κB signaling, executed by IKKB, and the kinetics of the NF-κB inhibitors (p105) (85, 99).

This clearly indicates, that the proposed IRF–GRN is only a part of a much bigger and more complex network of interactions, and needs to be further extended to model comprehensively immune activation versus tolerance in LCs.

## GRNs in LC Development

The development, differentiation and activation of LC are complex with many pathways/programs contributing to controlling these processes. In agreement with the requirement by LCs for signals from the epidermis, transforming growth factor-β1 (TGF-β1) is one of the key regulators produced by keratinocytes to program LC development and homeostasis. Indeed, TGF-β1-deficient mice lack LCs, owing to a failure in LC differentiation, survival, or both (100–102). TGF-β1 and bone morphogenetic protein superfamily member, bone morphogenetic protein 7, are key epidermal signals maintaining the pool of immature LCs in the epidermis (15, 16, 103). However, TGF-β signaling for ontogeny, homeostasis, and function of epidermal LCs does not follow a classical TGF-β signal transduction pathway, involving Smad3 as a transcriptional regulator (104).

Extensive recent research has shed light on a GRN underpinning TGF-β signaling, putting two transcription factors, PU.1 and ID2, in the center of the transcriptomic regulation of LC development. Chopin et al. (77) revealed that during *in vitro* bone marrow culture, *Runx3* is directly upregulated by PU.1. Functional importance of RUNX3 was further confirmed in that LC differentiation could be rescued by ectopic expression of RUNX3 in the absence of PU.1. Chopin and colleagues propose that RUNX3 is vital for mediating LC development, including restraining maturation, which is likely programmed by the TGF-β1-PU.1-RUNX3 transcription axis (105). Extending Chopin’s work, Zhang and colleagues proposed a collection of TFs and secondary regulators (106), including RUNX3 (107, 108) and STAT5 (109), as important for LC development and homeostasis. Complementing Chopin’s work on PU.1, counter-regulation between C/EBP and PU.1 might be necessary for LC development, in agreement with the concept of a GRN in which stimulatory and inhibitory interactions between transcriptional partners regulate the network. Mice with a dominant-negative C/EBP mutation completely switched myeloid cell fate from granulocytes/macrophages to LCs, lacking all other DC types. At the same time presence of wild-type C/EBP would completely block TNF-α-dependent LC development (110). Additionally, TGF-β signaling induces a member of the family of inhibitors of DNA binding proteins (ID2) critical for development of LC and cDCs, but not other DC types (77, 111, 112). Interestingly, while ID2 is indispensable for steady-state LCs, its role during inflammation remains variable, with only murine “long-term” inflammatory LCs stringently depended on ID2 (22, 105). Tissue-derived cytokines, such as TGF-β, play a critical role in shaping DC and macrophage differentiation and function across multiple tissue, including the gut and brain, as well as in the skin (16, 113–115). Interestingly, TGF-β signaling through RUNX3, working cooperatively with PU.1, key for LC development, seems to be uniquely important for the intestinal macrophage subset, which are constantly replenished from monocytes, both in the steady state and in inflammation (45, 114).

Biological networks have evolved to enable passing of biologically distinct information through shared channels [(“functional pleiotropism”) of signaling networks] (116). Indeed, in multiple DC types, IRF–GRN has been reported to orchestrate both cell development and function (72, 74, 75, 82, 117). However, in LCs, stark dichotomy seems to exist between developmental and activatory transcriptomic networks. Signaling of many of the LC key developmental molecules, such as TGF-β described above, or aryl hydrocarbon receptor, depend on PU.1 (77, 94, 118). While PU.1 is one of the core members of the cDC IRF–GRN, creating a transcriptional complex with IRF4 and/or IRF8 (119–122) in LCs, its function is apparently dissociated from these two key members of IRF family. Genetic analysis of human primary immunodeficiencies demonstrated that mutations affecting IRF8 transcriptional activity did not affect LC frequency, despite causing complete depletion of circulating monocytes and cDC (123). In concordance, investigations of *Irf4, Irf8*, and *RelB* k/o mice (77, 81, 105) did not detect alterations in LC presence and frequency in the epidermis, thus postulated these TF to be redundant/not important for LCs development. In contrast, transcripts for *IRF1, 4*, and *8* were either expressed at very high levels, or strongly induced by TNF-α signaling in human migratory LCs, creating the core or the LC GRN (13). This dissociation strongly suggests a dramatic modification of LC biology during transformation from a steady state to that of an activated cell, and further supports the argument of distinct LC ontogeny.

## LCs are Programmed by the Epidermis

Langerhans cells populate the epidermis from the early developmental stage as a dense network of immune system sentinels. These cells act as the outermost guard of the cutaneous immune system and are likely to induce the first reactions against pathogens encountered *via* the skin. A significant amount of research investigating LC biology in a variety of model systems has shown conflicting results. Recent development of “omics” technologies enabling investigations of human LCs at the level of the whole transcriptome has shed new light onto these long-standing questions.

Research elucidating LC ontogeny seems to have reached a consensus with evidence suggesting two waves of LC populating the epidermis in prenatal life, followed by the possibility of infiltration by inflammatory LCs reconstituted from blood progenitors (124–126). However, the place of LCs in the spectrum of innate immune cells is not clear. Whole transcriptome analyses indicate that at the gene expression level, as well as phenotypically and functionally, LCs are professional antigen-presenting cells, in the steady state distinctively different from both macrophages and cDCs. Even though it has been postulated that the LC transcriptome may reflect a macrophage-like origin of these cells (124, 127), none of the presented analyses supported this statement at the transcriptome level. This may be a reflection of the life-long programming exerted on the LCs by the epidermal environment, delivering homeostatic signals, such as TGF-β. Direct tracking of LC development *in vivo*, from the yolk sac progenitor through early skin populating cells, may shed more light on the exact moment when the LC acquire their unique characteristics, but the current evidence suggests strongly that LCs are programmed by the interactions with the epidermal microenvironment.

Studies following murine macrophage transcriptome and chromatin landscape across tissues, to distil the effect of ontogeny versus residence, clearly point to the profound effect tissue microenvironment takes in shaping the biology of tissue-resident cells (45, 46). Distinct, tissue-specific genetic programs can be clearly identified for all studied macrophage populations, irrespectively from the shared embryonic origin. It has been hypothesized that the tissue microenvironment coordinates chromatin binding of common and subset defining transcription factors, driving the changes in cell biology at a level of enhancer landscape and cooperative action of transcription factors (45, 46). This mechanism is likely to be similar in LCs, where epidermal cytokines could fine-tune the prototypic embryonic precursor GRN, programming LCs for their immune function.

Antigen uptake and sequestration by steady-state LCs can play a critical role in preventing viral infections (52, 53). On encounter with a danger signal, environmental and microenvironmental cues induce in LCs a switch between metabolic and immunological programs, resulting in extremely efficient antigen processing and presentation (12). Hence, on activation, LCs become more “DC-like,” specializing in activation of cellular adaptive immune responses. Characteristically, however, LC transcriptomic networks remain significantly different from the activated DC, remaining relatively stable, even following activation by pro-inflammatory cytokines. This may reflect a key evolutionary need related to tissue localization, preventing initiation of strong inflammatory reactions by LCs, most probably due to the importance of tolerance maintenance in the epidermal surface constantly exposed to the environmental insults.

Understanding of LC biology in the steady state gives clues to their baseline state and activation potential. However, in disease, or inflammation, the steady state of tissue and resident immune system cells is profoundly altered by the disease pathology. The chronic inflammatory process leads to the establishment of a disease-specific equilibrium, determining the mode of the response of the tissue-residing immune cells (27). However scarce, the investigations of the influence of the epidermal signaling on LC transcriptional programming strongly suggests that the key networks underpinning LC biology are fine-tuned by the epidermal microenvironment (13, 77). While TGF-β signaling mediated by PU.1/C/EBP seems to prevent LC activation, the effect of a high epidermal concentration of pro-inflammatory cytokines, such as TNF-α and TSLP, delivers an immune activatory switch to IRF–GRN operating in many DC populations. Therefore, even though LC may be functionally similar to cDCs, and despite sharing parts of the “hard-wiring” of GRN with DC and macrophages, the behavior of LC GRN seems to be distinctively different both in the steady state and upon activation, highlighting once more the uniqueness of LC transcriptomic programs and mechanisms of regulation mediated by the epidermis (Figure 5).

The findings reporting the role of transcription factors in LC are often controversial, or specific to a particular population/developmental stage, chiefly due to the challenges associated with the “*in situ*” analysis, and the paucity of suitable *in vitro* models. To further our understanding of the diversity of the programs and the regulation dynamics, more research is needed to elucidate the transcriptomics and GRN in steady-state LCs, especially in human skin. Furthermore, transcriptomics is capturing only one level of cell activation status. To fully elucidate the mechanisms regulating transcriptomic programs underpinning human LC function, investigations of the chromatin organization, histone modifications, protein translation and phosphorylation, at the level of isolated population, whole tissue, or single cell, need to be undertaken.

## Author Contributions

KC and JD reviewed and performed meta-analysis of publications regarding LC origin and development. AV and SS-B reviewed and performed meta-analysis of publications regarding LC maturation and activation. MP supervised the analysis and wrote the manuscript.

## Conflict of Interest Statement

The authors declare that the research was conducted in the absence of any commercial or financial relationships that could be construed as a potential conflict of interest.
